# Screening for Autism Spectrum Disorders - Validation of the Portuguese Version of the Social Communication Questionnaire

**DOI:** 10.1007/s10578-023-01535-8

**Published:** 2023-04-20

**Authors:** Manuela Araújo, Joana Calejo Jorge, Maria do Carmo Santos, Estela Vilhena, Pedro Oliveira, Paula Pinto Freitas

**Affiliations:** 1 Departamento de Psiquiatria da Infância e Adolescência – Centro Hospitalar Universitário do Porto, Porto, Portugal; 2 2Ai – School of Technology, IPCA, Barcelos, Portugal; 3 LASI – Associate Laboratory of Intelligent Systems, Guimarães, Portugal; 4 Instituto Ciências Biomédicas Abel Salazar-Universidade do Porto, Porto, Portugal; 5 EPIUnit, Instituto de Saúde Pública da Universidade do Porto, Porto, Portugal; 6 Center for Health Technology and Services Research (CINTESIS.ICBAS), Rua de Jorge Viterbo Ferreira, 228, Porto, 4050-313 Portugal

**Keywords:** Social Communication Questionnaire (SCQ), Autism Spectrum Disorders (ASD), Reliability, Validity, Screening

## Abstract

There are no assessment and screening tools for Autism Spectrum Disorders (ASD) validated for the Portuguese population. The Social Communication Questionnaire (SCQ) is an useful screening tool of ASD diagnosis. The main objectives of our study were to produce a Portuguese version of the SCQ (SCQ-PF), study its internal consistency, sensitivity and specificity in order to evaluate its validity as a screening instrument for ASD. We also wanted to study the impact of intellectual disability and verbal impairment and other mental disorders on SCQ-PF psychometric properties. The study included 211 children and adolescents, aged 4–17, divided in three groups: ASD Group (n = 96), Other Mental Disorders Group (OMD) (n = 63) and No Mental Disorders (NMD) Group (n = 52). Parents or other primary caregiver provided information on the SCQ items. The SCQ-PF score was significantly higher in the ASD group than in the other groups (p < 0.001). As to internal consistency, Cronbach’s alpha was 87%. ASD subjects were distinguished from subjects without ASD (OMD and NMD Groups) and the area under the curve (AUC) was 0.897 (95% Confidence Interval: 0.852–0.943), for a cutoff of 14, which yielded the highest AUC, with values of sensitivity and specificity 0.76 and 0.93, respectively. These findings show that SCQ- PF with a cutoff of 14 is an acceptable and useful screening tool for ASD in the Portuguese population.

## Introduction

Autism spectrum disorders (ASD) are lifelong neurodevelopmental disorders characterized by impaired social communication combined with restrictive and repetitive patterns of behavior and interests or activities [1]. Based on epidemiological studies conducted over the past 50 years, the prevalence of ASD appears to be increasing globally. The Centers for Disease Control and Prevention (CDC) estimated that one in 54 children (1.85%) among multiple communities in the United States have autism spectrum disorder (ASD). This new estimate is approximately 15% higher than two previous estimates of 2014 and 2016 [2]. There are many possible explanations for this apparent increase, including improved awareness, expansion of diagnostic criteria, better diagnostic tools and improved reporting [3, 4]. Early diagnosis and implementation of subsequent intervention confers a better developmental outcome; therefore, appropriate screening scales are of vital importance for assessment of children and earlier identification. Although routine developmental and autism screening is recommended by the American Academy of Pediatrics and the Centers for Disease Control and Prevention at ages 18 and 24 months, only a minority of children with ASD are identified by age 3 years, and many are identified after they enter school [5]. This emphasizes the importance of ASD screening at older ages, ideally involving long-term observation by different observers [6]. At the time of the writing, and to the best of the authors knowledge, there are no ASD screening instruments validated for the Portuguese population, which justifies the need of the development of such a tool. Therefore, this study aims to provide an useful instrument to address this need, since the Social Communication Questionnaire (SCQ), has been reported to be particularly adequate to use at older ages [7, 8].

The Social Communication Questionnaire (SCQ), previously known as the Autism Screening Questionnaire, has shown promising features as a screening measure for Autism Spectrum Disorders (ASD). SCQ is a screening tool developed by Rutter, Bailey and Lord [9] for autistic spectrum disorder in children and is derived from the version of the Autism Diagnostic Interview-Revised (ADI-R) [10] which is accepted as the gold standard for diagnosing autism. The ADI-R is based on the ICD-10 (International Classification of mental and behavioral disorders, World Health Organization, 1992) and DSM-IV (1994) diagnosis of Autism.

SCQ provides an operational diagnosis, which is based on behavioral item scores in three areas of functioning: reciprocal social interaction, language and communication and repetitive and stereotyped patterns of behavior.

There are two forms of the SCQ, the *Current* one assesses the child’s behavior at the time of the assessment and the preceding 3 months and the *Lifetime* one assesses symptoms throughout the child’s life and also contains a series of questions about the child’s behavior at age 4–5 years, the latter being the most used for diagnostic purposes.

At the initial validation study, that included 200 children and adults (age range 4–40), SCQ discriminated well between cases with and without ASD with a sensitivity of 0.85 and a specificity of 0.75 [11], with a recommended cutoff score for ASD of ≥ 15. SCQ primarily appeared as a screening instrument in research, indicating further need for clinical evaluation, since its lack of validation in a large community population sample [12]. Since it first became available it has been widely used and studied. SCQ has now been translated to several languages and its psychometric properties been evaluated in different countries and settings, consistently showing satisfactory psychometric properties (e.g.: Turkish: Avcil et al., 2015 [13]; Arabic: Aldosari et al., 2019 [14]; Simplified Chinese: Liu et al., 2022 [15]; Greek: Karaninis et al., 2022 [16]), although optimal cutoff values may need to be adjusted according to age or purpose [17].Although SCQ derived from ADI-R, which was designed for children above age 4 years, its authors suggest it can be applied to younger children provided their mental age exceeds 2 years [10]. However, caution must be taken about using SCQ under the age of 4 years, as literature has shown it is not age neutral [7, 8]. SCQ discriminatory power in youngest children appears to be reduced, with a sensitivity of 0.71 and a specificity of 0.54 [18]. For this reason, it was suggested that a lower cut-off point of 11 would be used in children aged less than 8 years [17, 18]. A recent large-scale (n = 819) study [15] performed in Chinese children aged 2–12 years from both general and clinical populations has provided different cutoffs according to age or specific diagnostic purpose: when distinguishing ASD cases from typically developing children a cutoff of 11 for children under 4 years and a cutoff of 12 for 4 years and above; when differentiating from other neurodevelopmental disorders, a cutoff of 14.

Changes in ASD prevalence have also been accompanied by changes in the prevalence of co-morbidities, such as intellectual disabilities [19] which are also related to language impairment and are frequent in other developmental disorders. In the validation study [11], the authors concluded that a cut-off score of 15 could be used for verbal and non-verbal subjects without impacting the psychometric properties. However, Eaves et al. [18] found differences between scores in verbal and non-verbal individuals related to missing data. Chandler et al. [20] reported similar SCQ scores in the high and the low IQ subgroups with a correlation between SCQ score and IQ close to zero. In contrast, the study by Eaves et al. [18] reported that SCQ scores were negatively correlated with IQ.

Despite the concerns that have risen about inconsistencies in its accuracy, a meta-analysis has concluded it is an acceptable screening instrument for ASD [8]. That study showed that methodological variations were responsible for loss in accuracy, namely: use of the *Current* version instead of the *Lifetime* version of the SCQ; use of the SCQ under 4 years of age and application of the SCQ to convenience samples instead of community samples [8].

The authors intend to produce a Portuguese version of the Lifetime form - Social Communication Questionnaire- Portuguese Form (SCQ-PF) and evaluate its validity as a screening instrument for ASD in the Portuguese population, by examining its internal consistency, sensitivity and specificity (comparing SCQ-PF scores between different groups: ASD children, children with other mental disorders and children with no mental disorders). Bearing in mind that research has shown that SCQ’s sensitivity-specificity ratio is worse when distinguishing ASD from other developmental disorders [21] in which intellectual disability and verbal impairment often occur, authors also intend to examine how these may affect SCQ scores.

## Methods

The study was approved by the Hospital’s Ethical Committee. Written informed consent was obtained for every participant in the study, who was handed a printed information about study design and procedures and authors’ contacts in case of withdrawal, assuring there would be no interference with clinical care in such case.

Permission was obtained from SCQ’s legal owner of rights (Western Psychological Services) in order to use the instrument and perform the study.

### Development of the SCQ-Portuguese Form

SCQ is a brief questionnaire, with 40 questions per form, with “yes” or “no” responses. It should take approximately 10 min to complete. The first question evaluates the verbal ability of the subject (presence of phrase speech) and it does not have scoring value. To subjects who are considered non-verbal (answer 0 in question 1) questions 2–7 do not apply. Each item is scored 0 or 1 and the sum of the 39 items yields a total score (ranges from 0 to 39). The scale was translated by 2 of the authors and then reviewed by 2 other mental health professionals. All of them were child and adolescent psychiatrists, experts on autism spectrum disorders. When necessary, items were culturally adapted, such as item nr. 34 (spontaneous joining in social games) in which the English-culture rhymes were replaced by Portuguese-culture ones. The questionnaire was then back translated by an independent bilingual translator who had no previous knowledge of the questionnaire. For example: item 2 in the original is “Do you have a to and fro “conversation” with her/him that involves taking turns or building on what you have said?” was translated to “Consegue ter um diálogo com ele/a em que fale um de cada vez (alternadamente) ou em que ele/a continue a falar a partir do que a outra pessoa disse?” and then backtranslated to “Are you able to have a back and forth conversation with him/her where each of you speaks in turns or where he/she continues to speak based on what the other person said?” A pretest of the final translated SCQ was evaluated by giving it to a sample of 10 parents – a convenience sample recruited amongst Hospital workers. The parents were asked to answer to the Portuguese version of the questionnaire and to identify any difficulties in understanding the questions. Next, minor language issues were resolved by consensus amongst all of the authors, which led to the final version of the Portuguese form of the Social Communication Questionnaire (SCQ-PF).

### Sample and Data Collected

The sample was constituted by three groups of children and adolescents, aged 4–17. Group one (G1) was a sample of children and adolescents who had been referred for assessment for a suspicion of ASD (considered a “high-risk” group). Group 2 (G2) was a sample of children and adolescents who were being followed due to another psychiatric diagnosis. Group 3 (G3) was a convenience sample of children and adolescents from the general population who did not present any known current mental health or learning disorder. Group one (G1) was recruited at the Child and Adolescent Mental Health Department of Centro Hospitalar Universitário do Porto (CHUP), which is a tertiary university hospital and at the Portuguese Association for Developmental Disorders and Autism-North (APPDA-Norte). Group two (G2) was recruited at the Child and Adolescent Mental Health Center of CHP. We selected G2 and G3 participants controlling age and gender to ensure homogeneity with G1.

All of the caregivers of every child and adolescent who were invited and accepted to participate were given a printed SCQ-PF copy which was answered in writing and handed back to one of the authors. An interview was then conducted by a child psychiatrist (one of the authors) with at least one significant caregiver of the child (one of the parents or legal tutors). Sociodemographic data was obtained and a short clinical interview based on a Portuguese version of the Kiddie Schedule for Affective Disorders and Schizophrenia (K-SADS) [22] was performed. Kiddie-SADS is a semi-structured interview designed to diagnose childhood mental disorders and thus allowed the authors to identify any suspicion of mental disorders that should then be submitted to full clinical psychiatric assessment. These above-mentioned data were collected for all participants. G1 and G2 subjects’ clinical records were consulted in order to obtain information regarding previous psychiatric diagnosis. Standardized developmental/cognitive assessment and full autism-specific assessments were performed to all G1. This complete clinical evaluation would also be performed to G2 and G3 subjects, in case of any suspicion of ASD identified at the clinical interview. The developmental/cognitive assessment was performed with one of the following instruments (according to the age, clinical characteristics and cooperation level of the child): Vineland Adaptative Behavior Scales, Raven Progressive Matrices, Griffiths Mental Development Scales, Weschler Preschool and Primary Scale of Intelligence, or Weschler Intelligence Scale for Children. Results of this assessment were converted in a qualitative classification of the child as either presenting (or not) an Intellectual Disability (ID). Autism-Specific Assessment included use of the Autism Diagnostic Interview – Revised (ADI-R) [10, 23]and the Autism Diagnostic Observation Schedule (ADOS)[24]. When used together, this diagnostic method is considered gold standard in the assessment and diagnosis of ASD [25]. The authors that assessed children with ADI-R and ADOS are Child and Adolescent Psychiatrists with certified training in these instruments. The ADI-R is a standardized, semi-structured, investigator-based interview for caregivers of persons suspected of having ASD. Using the ADI-R, the trained interviewer evaluates three functional domains: language and communication; reciprocal social interaction; and restrictive, repetitive and stereotyped behaviors and interests based on parents’ responses to open-ended questions. The ADOS is a semi-structured, standardized assessment of social interaction, communication, play, and imaginative use of materials for individuals suspected of having ASD. The observational schedule is conducted by a trained interviewer and consists of four 40–60-minute modules, each designed to be administered to different individuals according to their level of expressive language. In cases of discrepancy between these 2 instruments, a panel of 4 child and adolescent psychiatrists reviewed the case and reached a final diagnosis, based on the DSM-5 criteria for Autism Spectrum Disorders. Once the evaluation was completed, the participants were reclassified into 3 “final” groups: an “ASD Group”, an “Other Mental Disorders (OMD) Group” and a “No Mental Disorders (NMD) Group”.

### Statistical Analysis

Descriptive statistics were calculated and expressed. To examine differences between groups, conventional statistical tests were used to compare continuous (student’s t-tests, one way analysis of variance (ANOVA)) and categorical (chi-square and Fisher’s test) variables. SCQ-PF’s internal consistency was measured with Cronbach’s alpha coefficient. Results of the SCQ-PF were compared to the results of the gold standard assessment. A receiver operating (ROC) analysis was performed to assess the discriminant power of the SCQ in distinguishing ASD cases from cases without ASD, and to estimate sensitivity and specificity.

Confirmatory factor analysis (CFA) was used to test how well the measured variables represent the factor structure of the SCQ. Four continuous latent variables were regressed (social, communication, abnormal language, stereotyped behavior). The goodness of fit for each factor structure was evaluated using several descriptive criteria: the ratio between Chi Square and degrees of freedom (${\chi }^{2}/df$), the Comparative Fit Index (CFI), the Tucker-Lewis index (TLI) and the Root Mean Square Error of Approximation (RMSEA) with its 90% confidence interval (RMSEA 90% CI) [26, 27]. For each variable, the magnitudes of factor loadings were considered. Variables with a factor loading of 0.3 or greater were considered representative of the construct being measured in each domain. The level of statistical significance was set at *p* < 0.05. Statistical analysis was performed in AMOS and SPSS (IBM Corp.).

## Results

**Fig. 1:**
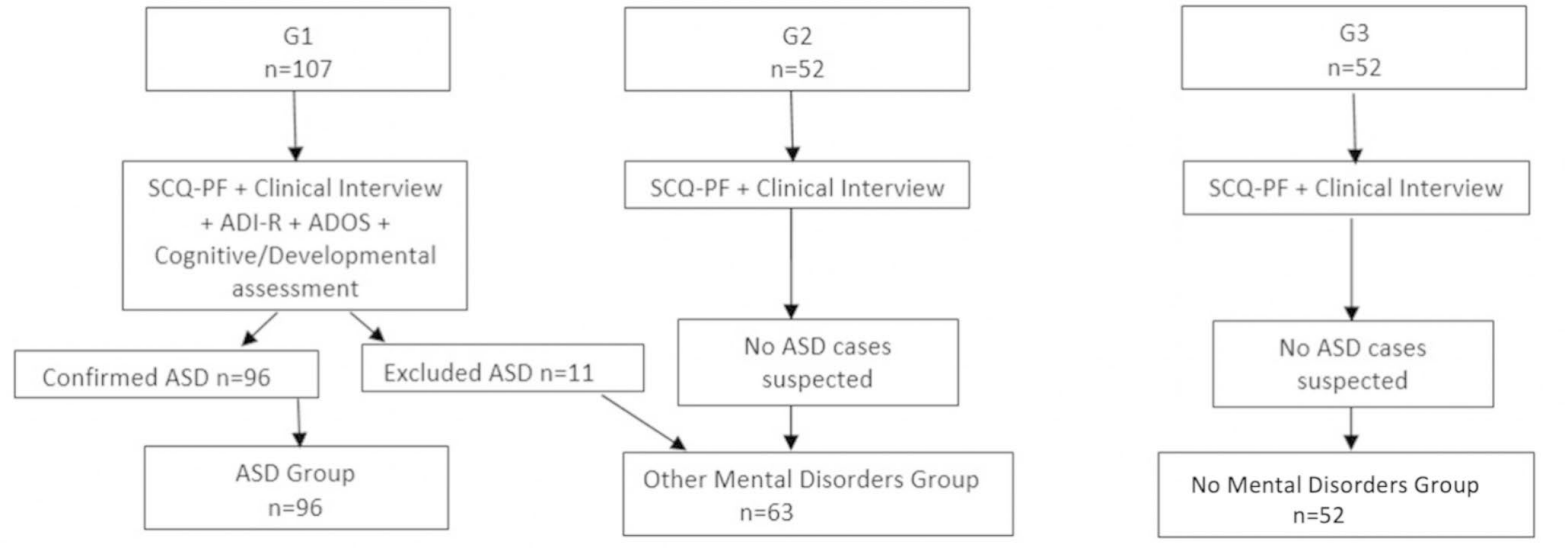
Procedures and groups constitution

The sample was constituted by a total of 211 children and adolescents, distributed in 3 initial groups: G1 had 107 participants; G2 and G3 had 52 participants each. After completing the clinical assessment, participants were reclassified into 3 final groups: a group comprising participants in which an ASD diagnosis was reached (the ASD group) a group with participants with Other Mental Disorders (OMD) and group of participants with No Mental Disorders (NMD). An ASD diagnosis was excluded in 11 G1 participants who were transferred to the OMD Group. NoASD diagnosis arouse in NMD Group (n = 52). In the “Other Mental Disorders Group”, 25 participants (39.7%) had received an ADHD diagnosis, 9 participants (14.3%) an Anxiety Disorder diagnosis, 7 participants had Behavioral Disorders and 6 participants had Specific Learning Disorders. All others diagnosis were scarcely represented with only one subject per diagnosis. The SCQ-PF ability in differentiating ASD from no-ASD cases was evaluated according with the final diagnostic classification: ASD, OMD and NMD. Age distribution was similar between groups: in the ASD Group the mean age was 8.38 years (± 3.4), in the OMD Group the mean age was 9.33 years (± 3.8) and in NMD Group the mean age was 9.37 (± 3.9). In terms of gender there was a clear predominance of boys: 74 (77.1%) in the ASD Group, 48 (76.2%) in the OMD Group and 33 (63.5%) in the NMD Group. Demographics are presented in Table 1. Procedures and groups constitution are presented in Fig. 1.

**Table 1:** Descriptive Statistics

Groups (n)	ASD (96)	OMD (63)	NMD (52)	
Sex (n, % male)	74 (77.1)	48(76.2)	23 (63.5)	*p* = 0.169
Age [mean (sd)]	8.38 (3.4)	9.33(3.8)	9.37 (3.9)	*p* = 0.163
Intellectual Disability (n,%)	60 (62.5)	13 (21.6)	0 (0)	*p* < 0.001
Verbal Impairment	30(31.3)	2(3.2)	0(0)	*p* < 0.001
Carer’s Education (n,%)				*p* < 0.001
≤ 4 years	2 (2.1)	0	4 (7.8)	
5–12 years	67 (71.3)	48 (82.8)	16 (31.4)	
> 12 years	25 (26.6)	10 (17.2)	31 (60.8)	
Carer’s Profession (n,%)				*p* < 0.001
Workers	17 (18.9)	16 (27.1)	3 (5.9)	
Technicians	52 (57.8)	33 (55.9)	20 (39.2)	
Professionals	21 (23.3)	10 (16.9)	28 (54.9)	
Carer’s Employment status (n,%)				*p* < 0.001
Both unemployed	5 (5.4)	2 (3.4)	11 (21.6)	
One unemployed	48 (51.6)	27 (45.8)	7 (13.7)	
Both employed	40 (43.0)	30 (50.8)	33 (64.7)	

ASD-Autism Spectrum Disorders; OMD-Other Mental Disorders; NMD-No Mental Disorders

### SCQ-PF’s Internal Consistency, SCQ-PF Total Scores and Subscores

The SCQ-PF’s internal consistency was evaluated calculating Cronbach’s alpha. For the total SCQ-PF score (when considering all 39 items) Cronbach’s alpha was 0.873. This estimate excluded 32 participants to whom questions 2–7 do not apply because they were non-verbal subjects. When considering only items 8–40, all respondents (211) were included and Cronbach’s alpha was re-estimated to be 0.899.

**Fig. 2:**
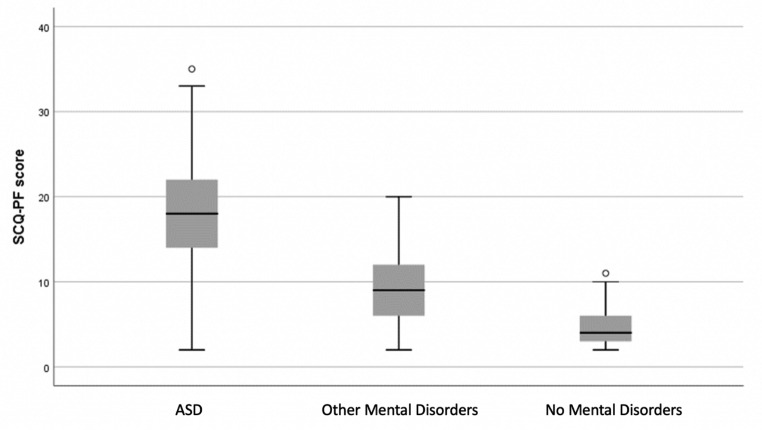
SCQ-PF scores comparison between groups

The total SCQ-PF mean score was compared between groups. There was a statistically significant difference between groups as determined by one-way ANOVA (_F2.208_=116.9, *p* < 0.001). A Turkey post hoc comparison test revealed that the SCQ-PF score was statistically significantly higher in the ASD group (17.63 ± 6.8) than in both the OMD Group (9.25 ± 4.0) and the NMD group (4.67 ± 2.2), *p* < 0.001. The difference between the OMD Group and the NMD Group is also statistically significant (*p* < 0.001). The SCQ-PF score did not vary by gender or age and there was no interaction between gender and age group (*p* > 0.005). SCQ-PF scores comparison between groups are presented in Fig. 2.

The total SCQ-PF score was computed considering items 2–40 to verbal subjects and items 8–40 to non-verbal subjects. A second non-verbal score was computed to all participants considering only items 8 to 40. These two scores correlate highly (*r*_*s*_=0.978, p < 0.01) which indicates no need to adjust for uneven number of items in verbal and non-verbal subjects. Considering SCQ’s clinical domains, three subscores were considered: social interaction subscore, communication subscore and repetitive behaviours subscore. For the overall sample (n = 211) there was a higher correlation between the social interaction and communication subscores (*r*_*s*_=0.666; *p* < 0.01); than between communication and repetitive behaviours subscores (*r*_*s*_=0.587; *p* < 0.01) and than between social interaction and repetitive behaviours subscores (*r*_*s*_=0.536; *p* < 0.01). This pattern repeats when considering the ASD subsample only (n = 96), although with weaker levels of association: the social interaction and communication subscores correlate higher (*r*_*s*_=0.498; *p* < 0.01) than between communication and repetitive behaviours subscores (*r*_*s*_=0.393; *p* < 0.01) and between social interaction and repetitive behaviours subscores (*r*_*s*_=0.263; *p* < 0.01).

The results of the CFA revealed a 4-factor model of the SCQ, latent variables (social, communication, abnormal language, stereotyped behavior), provided a good model fit for the data. As recommended by Brown [28], the model is considered to have “adequate fit” if the RMSEA is less than 0.08 and the CFI is greater than 0.9; “good fit” is indicated by an RMSEA less than 0.05, a CFI greater than 0.95 and ${\chi }^{2}/df$ values less than 3.All items significantly loaded their hypothesized factors and adequate fit indices, ${\chi }^{2}$=1092.97; $df$=655; p < 0.001; ${\chi }^{2}/df$=1.67; CFI = 0.8; TLI=0.8; RMSEA = 0.061; 90%CI =[0.055, 0.068].

### Discriminant Validity

A receiver operating characteristic (ROC) analysis was used to assess the discriminant power of the SCQ-PF in distinguishing ASD cases from no-ASD cases. The area under the curve (AUC) was 0.897 (95% Confidence Interval (CI): 0.852–0.943), for a cutoff of 14, which yielded the highest AUC. For the established cutoff of 15, sensitivity was 0.73 and specificity was 0.95. When considering a cutoff of 14 the values of sensitivity and specificity were 0.76 and 0.93, respectively.

**Fig. 3:**
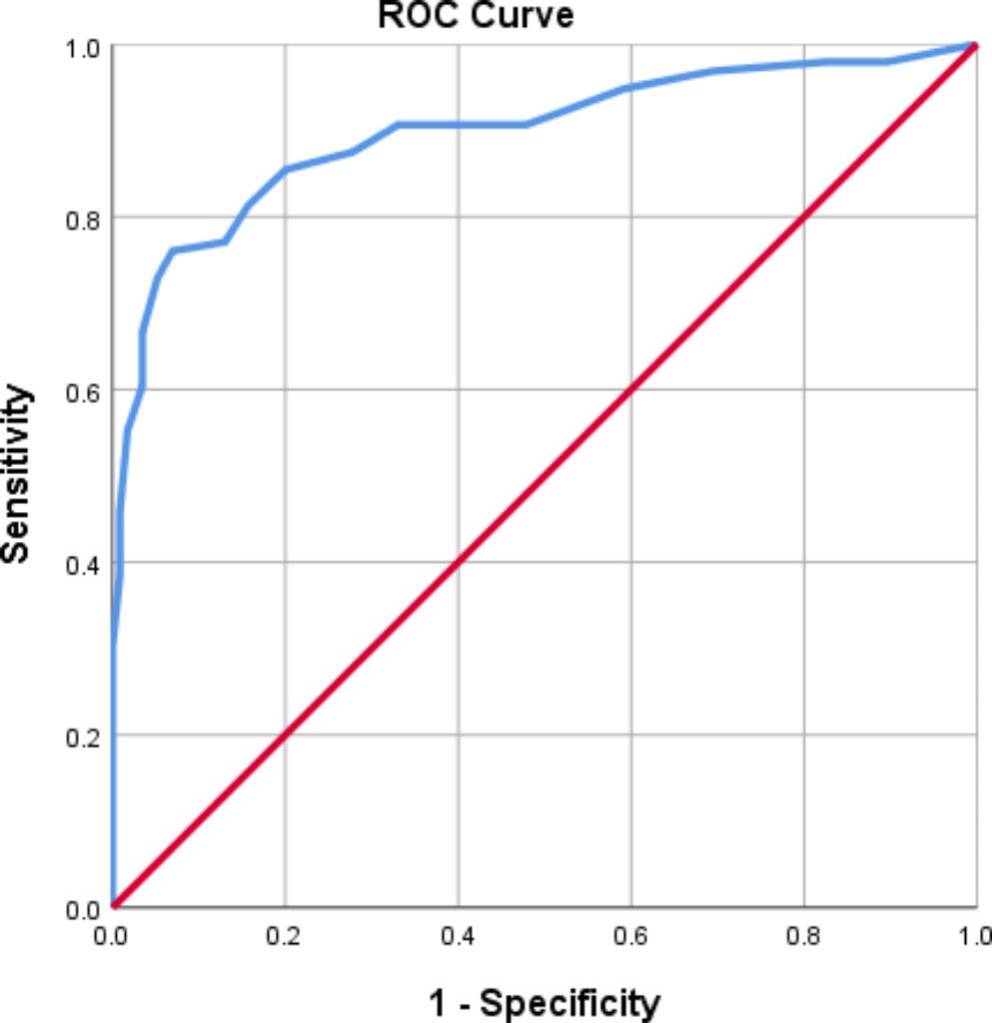
Discriminant validity of the Portuguese Form of the Social Communication Questionnaire (SCQ-PF) total score in the receiver operating characteristic (ROC) curve analysis (n = 211)

Considering SCQ-PF results for the subscores, the AUC were 0.804 for the communication score (95% CI: 0.742–0.865), 0.830 (95% CI: 0.776–0.885) for the repetitive behaviors score and 0.875 (95% Confidence Interval (CI): 0.824–0.926) for the social interaction score. Figure 3 presents the Discriminant validity of the Portuguese Form of the Social Communication Questionnaire (SCQ-PF) total score in the receiver operating characteristic (ROC) curve analysis (n = 211).

### Intellectual Disability Effects and Verbal Impairment

The ASD Group has a predominance of participants (*n* = 60, 62.5%) with an Intellectual Disability (ID), while in the OMD Group these participants represent 20.6% (*n* = 13). There are none intellectually disabled participants in the NMD Group. Comparing SCQ-PF scores between ID and no-ID subjects, in both ASD and OMD Groups we found that SCQ-PF scores higher when ID is present as illustrated in Table 2.

**Table 2:** SCQ-PF mean scores in participants with and without Intellectual Disability

Final groups	Intellectual Disability *n* (%) SCQ-PF mean score		No Intellectual Disability *n* (%) SCQ-PF mean score	
ASD	60 (62.5%)	18.9 (± 6.4)	36 (37.5%)	15.6 (± 7.0)
OMD	13 (20.6%)	11.0 (± 3.2)	50 (79.4%)	8.8 (± 4.1)

ASD-Autism Spectrum Disorders; OMD-Other Mental Disorders

A logistic regression analysis concluded that ASD subjects have approximately a 42 times higher chance of having a SCQ-PF positive result than no-ASD subjects (Odds Ratio (OR) 42.45, 95% Confidence Interval (CI) [18.00-100.09]). Having an ID also increases the probability of having a positive result in the SCQ-PF, but at a much lower extent (OR 8.35,95% CI [4.39–15.88]). When considering both variables together in the model (ASD and ID), ASD increases the probability of a SCQ-PF positive result approximately 30 times (OR 29.63,95% CI [11.99–73.25]) and ID approximately 2 times (OR 2.37, 95% CI [1.02–4.49]). There is no interaction (ID and positive ASD diagnosis both contribute to raise the risk of a SCQ positive result although independently).

Within all participants, verbal impairment was present in 32 subjects (were classified as non-verbal because the answer to SCQ-PF’s item 1 was 0), 30 of whom belonged to the ASD Group (there were only 2 non-verbal subjects out of the ASD Group). This means that approximately 1/3 of the participants in the ASD group (n = 96) were non-verbal (n = 30). These 30 subjects have a mean total SCQ score of 20.97 (± 5.3). The other 66 ASD subjects (verbal) have a mean total SCQ score of 16.11 (± 6.86), which is significantly lower (*p* < 0.05). Therefore, ASD non-verbal subjects score higher in the SCQ-PF than verbal subjects. In the non-verbal ASD subjects, SCQ-PF total score was below the suggested cutoff of 14 (i.e. false negatives) in 2 cases (6.7%) whereas in the verbal ASD subjects the SCQ-PF was below 14 in 21 cases (31.8%). Considering all non-verbal subjects (n = 32) a ROC analysis was performed which yielded a suggested cutoff of 16, however, it should be reminded that there were only 2 non-verbal no-ASD subjects.

## Discussion

Our study is the first to assess the use of a Portuguese SCQ screening tool in Portugal’s population with 10.29 million individuals [29]. The SCQ-PF showed a high internal consistency coefficient as measured by Cronbach’s alpha coefficient for the total SCQ-PF score (0.873) which was comparable to other SCQ validation studies [13–16]. This is a good feature found in SCQ-PF which revealed a reliable instrument delivering consistent scores.

The initial SCQ validation study [9] showed a sensitivity of 0.85 and a specificity of 0.75 for the cut-off of 15. Our study, compared with the initial study, has a lower sensitivity, 0.73, and a higher specificity, 0.95, for the same cut-off. Other studies showed values of sensitivity and specificity similar to the initial validation study, including the Turkish version of SCQ (sensitivity of 0.94 and specificity of 0.84) [13]. The Arabic version of SCQ showed more comparable values to our study, with lower sensitivity, 0.80, and higher specificity, 0.97 [14]. We observed that using a lower cut-off of 14, the sensitivity was raised to 0.76 and the specificity decreased to 0.93. Because of this better performance, in particularly with respect to sensitivity, we recommend the use of the established cut-off value of 14 for the Portuguese version of the SCQ. These values of sensitivity and specificity are considered acceptable for developmental screening tests. The very good specificity provides the possibility of differentiating those with ASD from those that exhibit some signs typical of ASD but have not the disorder and can strengthen the SCQ-PF as a screening instrument for high-risk groups. Also, higher specificity values can avoid costly referral for in-depth [30] which is an important feature in a middle-income country as Portugal. As suggested by other authors, the cut-off selected for use in clinical settings may differ according to age and purpose and should be adjusted per the user’s goals to prioritize sensitivity or specificity depending on goals [17, 21, 31].

In the original standardization study [11] empirical findings suggested that SCQ mean total scores were broadly comparable between ASD individuals with and without language, although the mean score for those without language was significantly lower. In this study, the contrary appears, being SCQ-PF results significantly higher in ASD non-verbal subjects than in verbal subjects. Different cut-off scores may be needed for verbal and non-verbal individuals since several items related to verbal language are not included in the final score for non-verbal individuals. In our study an adjusted cutoff of 16 was better in discriminating ASD in the non-verbal subjects; however, caution must be taken in interpreting this finding, due to extremely low number of non-verbal subjects other than in the ASD group (n = 2). It would be advisable to examine SCQ-PF results in a sample with a higher proportion of non-verbal no-ASD subjects. Since verbal impairment is also related to cognitive functioning this would also imply including more intellectual-disabled no-ASD patients. Due to our sampling procedures, children with ASD had mostly Intellectual Disability, whereas children with other mental disorder or from the general population mostly were without Intellectual Disability. It is therefore possible that the strong discriminant ability obtained with the SCQ to differentiate our two samples may have been slightly overestimated. However, the high levels of specificity found in this study should help maintain Good psychometric properties in a different sampling context. It would be interesting to replicate this study in samples of similar developmental level. SCQ-PF needs further study in no-ASD children with developmental problems.

Our study included older children and a clinical sample with considerable mental health difficulties, contrasting to most of the studies since the original validation study that compared children with ASD with children with a range of other developmental or behavioral difficulties [17, 32]. Decreased specificities have been found when heterogeneous psychiatric clinical control samples were evaluated [33]. In our study, although we did identify other mental health disorders, we did not discriminate the screen performance of SCQ-PF. In a future study of the SCQ-PF, it would be useful to discriminate the influence of these disorders on the SCQ score.

Our samples were recruited for convenience both in clinical and population samples. Results might be sensitive to some undocumented selection biases. However, the reasonable sample size and the recruitment across multiple sources should have protected our study against substantial biases and atypical findings. The absence of mental disorder or ASD among controls was evaluated by parental reports only in the clinical interview and no direct clinical evaluation of the child was performed unless suspicion had arisen. This may had contributed to parents missing symptoms of mental disorder or ASD among controls even though some research indicates that parents can be a reliable source of information regarding their child’s development [34]. Nevertheless, there is evidence of some discrepancies in parent’s reports of child’s mental health and development, specifically in different awareness to symptoms, with an increased sensitivity to child’s restricted interests and behaviors than to the social and communication challenges [35]. Given the demonstrated utility of parent concerns, it is important to examine how parents’ characteristics may influence their reported concerns. For example, parent’s socioeconomic status is often inversely associated to concerns about their child’s development [36]. Since caregivers of the NMD Group were highly educated, this may have strengthened our study preventing the expected discrepancies in parent’s reports thanks to the increasing of parents’ awareness about their child’s development. In a future study on the SCQ, it would be interesting to compare results on the SCQ score with results using the ADOS and ADI-R. Also, considering the new criteria of the DSM-5, it would be demanded an update of the SCQ as well as of other ASD screening instruments in order to comprehensively satisfy the new criteria of the DSM-5 [37].

### Summary

In this study, a relatively large and rather typical child and adolescent psychiatric sample was used to evaluate diagnostic validity, which lends additional support for the utility of the SCQ as a screener in clinical practice. Although the increasing awareness to early detection of ASD, there are still some missing or delayed diagnosis of ASD which underlines the importance of screening of ASD in later developmental phases. This study shows that the SCQ- PF with a cutoff value of 14 is a valuable screening tool in high-risk children aged 4–17 for correctly identifying those with a possible diagnosis of ASD compared with other developmental diagnoses.
